# Survey of inpatient satisfaction with integrated services in China, a retrospective study

**DOI:** 10.3389/fpubh.2026.1863748

**Published:** 2026-07-30

**Authors:** Fang Cao, Fuyuan Hong, Yiping Ruan, Liting Zhuang, Yueling Chen, Xuejing Chen, Miao Lin

**Affiliations:** 1 Department of Nephrology, Provincial Clinical Medical College of Fujian Medical University, Fujian Provincial Hospital, Fuzhou University Affiliated Provincial Hospital, Fuzhou, China; 2 Department of Nursing, Fuzhou University Affiliated Provincial Hospital, Fuzhou, China

**Keywords:** inpatient, integrated medical service, patient experience, patient satisfaction, structured interview

## Abstract

**Introduction:**

Patients' satisfaction with a hospital involves an evaluation based on comparison of the medical services provided by the hospital with patients' expectations in relation to their disease and quality of life. Deficiencies in quality can motivate medical institutions to conduct continuous improvement to meet patients' health needs. The importance of measuring and managing patient satisfaction has become increasingly apparent. Integrated Care is a concept bringing together inputs, delivery, management, and organization of services related to diagnosis, treatment, care, rehabilitation, and health promotion. Integration has been proposed as a means to improve the services satisfaction.

**Methods:**

The article retrospectively included data on satisfaction of patients discharged from hospitals between 2022 and 2023. This study interviewed a total of 43,693. The design of the interviews was based on the indicator database of patient experience from the National Medical Management Centre. The researchers identified three themes and 16 subthemes. Descriptive statistics (means, SDs, and *t*-tests) were used to summarize satisfaction scores and their annual changes across the 16 subthemes.

**Results:**

The overall scores of the interview topics in 2022 and 2023 were 97.32 ± 1.72 and 97.34 ± 3.49 (*P* = *0.887*), respectively. In the interviews about the patient experience in 2022 and 2023, the three lowest-ranked satisfaction scores were (87.16 ± 3.12 vs. 88.65 ± 4.82; *P* < *0.001*) for caregiver services, (93.02 ± 4.50 vs. 95.59 ± 3.88; *P* < *0.001*) for meals, and (94.97 ± 3.54 vs. 94.35 ± 4.05; *P* = *0.013*) for wards and bathrooms. The satisfaction survey of doctor's respect, listening, nurse's service, pain management, examination, flat car wheelchair were significantly higher in 2023 than in 2022 (*P* < 0.001). There were no significant differences regarding satisfaction survey on medication notification, health education, and road signs (*P* > 0.05).

**Conclusions:**

By focusing on patients' needs, emotions and experiences, we may provide comprehensive care and support and truly respond to the “patient-centred” concept of medical service, which may improve not only medical quality but also the doctor–patient relationship and promotes the development of the medical industry.

**Relevance to clinical practice:**

Due to human biological attributes, we will all face health problems in our life and play the role of “patients.” We are committed to providing patients with high-level medical services with convenient processes, efficient operation, and symmetric information.

## Background

According to data from the Chinese Statistics Bureau, the number of beds in medical institutions per 1,000 people increased from 2.49 in 2002 to 6.70 in 2021 (1). With sufficient medical resources, people prefer medical locations that provide a better experience under the same conditions.

The concept of patient experience should be clearly defined through performing a systematic integrative review (2). Patient experience is recognized as a means of assessing healthcare delivery with organizations in many countries (3, 4). As patients' expectations for medical quality continue to increase, domestic and international scholars have conducted in-depth studies on the patient experience. Improving patients' medical experience has attracted the attention of medical institutions and their administrators. Patients' satisfaction with a hospital involves an evaluation based on comparison of the medical services provided by the hospital with patients' expectations in relation to their disease and quality of life. Patient experience focuses on whether specific aspects of care occurred, while patient satisfaction gauges whether patient expectations were met (5). Patient satisfaction is determined by the patient experience. Therefore, the experience of patients and their families in the medical service process is quantified as patient satisfaction (6, 7). The evaluation of patient satisfaction based on patients' medical experience is an evaluation of the quality of the medical service. Despite controversy on this issue, patient satisfaction can be used as an indicator of the quality of medical care in a hospital (8–10).

Through humanistic care, medical institutions can provide good doctor–patient relationships and improve patients' trust. These aspects are critical for a smooth medical process and for continuous improvements in the quality of hospital medical services. Humanistic care can enhance communication and understanding and reduce conflicts between doctors and patients, and it can promote the establishment of harmonious doctor–patient relationships. Currently, both developing and developed countries attach great importance to the study of patient satisfaction. Compared with developed countries such as the United States, developing countries such as China focus on different research areas due to their different national conditions (11, 12). Studies of patient satisfaction in China started relatively late. Satisfaction surveys conducted by hospitals can conveniently collect information on patients' perceptions and experiences of medical quality during the diagnosis and treatment process and can help medical institutions identify shortcomings in medical services from the perspective of patients. Deficiencies in quality can motivate medical institutions to conduct continuous improvement to meet patients' health needs (13). The importance of measuring and managing patient satisfaction has become increasingly apparent (14).

Integrated Care is a concept of bringing together inputs, delivery, management, and organization of services related to diagnosis, treatment, care, rehabilitation, and health promotion (15). A general definition of Integration is the act of making a whole out of parts. Integration has been proposed as a means to improve the services satisfaction (15). As main healthcare providers, nurses make a significant impact on patients' perceptions about their satisfaction (16). In order to achieve the goal of high-care quality, decision makers should not only know whether a patient is satisfied but, more importantly, why the patient is not satisfied with the healthcare service. If healthcare organization managers are able to identify satisfaction, they could accordingly adjust the performance of services that they offer (17). Currently most studies about satisfaction of outpatients conducted in tertiary hospitals were of small sample size which only focused on service attitude but not diet, hospital supporting facilities, health education, and other contents (16, 18, 19). Therefore, it is necessary to carry out surveys in health services to constantly measure patients' satisfaction and learn about their expectations, suggestions and feedbacks, so as to guide healthcare workers as to which items should be prioritized and which require alterations in the service (1).

A cross-sectional survey was undertaken from 2022 to 2023 with discharged patients in Fujian Provincial Hospital. The data were collected by structured telephone interview. Interviews are a form of communication that takes place face-to-face or through other media, such as telephone or video. Interviews are used by researchers to obtain information, understand participants' viewpoints and discuss issues. Therefore, they are powerful data collection tools (20). In structured interviews, the interviewer uses a prepared list of questions and asks them in the same way in a specific order. This method is helpful for maintaining the consistency of the questions, and the questions are asked using the exact same wording to obtain responses from the other party. Telephone interviews can save time and costs and are often used in cross-regional or cross-border communications.

## Participants and methods

### Participants

Patients who were discharged from the investigator's hospital in 2022 and 2023 participated in this interview survey. The interviewees were divided according to the hospital departments, not by the type or severity of disease. Convenience sampling was used, and the patients were shuffled by a computer according to their discharge time. The inclusion criteria were as follows: (1) hospitalization for more than 24 h, (2) continuous interviews, and (3) discharge time of 3–7 days. The exclusion criteria were as follows: (1) patients who were automatically discharged from the hospital, (2) death, (3) medical disputes between patients and the hospital, (4) patients who did not participate in the caregiver service in the hospital, (5) patients in the cadre's ward, delivery ward, ICU, and day ward, (6) Wrong phone number, and (7) The patient did not answer the phone. The satisfaction survey sample size is calculated based on the confidence level and the margin of error, the confidence level (usually 95%) and the margin of error (usually ±3% or ±5%) of the hospital satisfaction survey in China. The annual discharge of the hospital where the author works is about 125,000. Based on these parameters, the sample size can be calculated using a statistical formula to get about 500 copies. This study employed a convenience sampling method, which may lead to selection bias. To mitigate the potential selection bias inherent in convenience sampling, the following measures were implemented. First, patients were randomly shuffled by a computer algorithm based on their discharge time before sampling, ensuring that no systematic preference was given to any particular patient subgroup. Second, the final sample of 43,693 patients represented 17.5% of the total 250,000 discharges over the 2-year period (125,000 per year), which is substantially larger than the minimum required sample size of 500 calculated using the standard formula (confidence level 95%, margin of error ±3%). This large sample size helps to offset the limitations of convenience sampling by increasing the precision of estimates and reducing random error.

At the beginning of the interview process, the researchers indicated the purpose and expected duration of the interview, solicited the wishes of the survey participants, and informed them of their rights. To ensure the independence of this survey, the computer system displayed the information of all patients who participated in this survey. Participants were also informed that they would not be asked for any personal information during the interview. They could refuse to participate or terminate their participation at any time without consequence. Ultimately, a total of 43,693 discharged patients were interviewed over 2 years.

### Methods

The article retrospectively compiles data on satisfaction of patients discharged from hospitals between 2022 and 2023.The data were collected by structured telephone interview, which might cause response bias as patients might provide answers that conform to social expectations. We have taken the following measures to reduce the bias of the interviewers. Before the official beginning of the survey, unified training was provided to the investigators. All investigators had certificates for professional nurse training qualification and more than 5 years of clinical nursing experience. We chose a quiet office without a flow of people to ensure that the data collection would not be affected by interruptions. The researchers were familiar with the data recording equipment and the interview questions. Before collecting the data through formal telephone interviews, the researchers conducted a pilot test on the interviewers and found no ambiguity in the wording. Before the formal interviews began, the investigators introduced themselves, solicited oral informed consent from the participants, and informed the participants of the purpose and duration of the interview in easily understood language for approximately 5–10 min to establish a good relationship with the respondents. After proposing the interview subthemes based on the interview outline, the researchers attempted to talk less and listen more and to remain open and honest. The researchers were ready to address unexpected emotions from the participants at any time. The researchers sometimes unintentionally invoked unpleasant experiences of the participants, such as pain, waiting, and the unknown. Once the data collection was complete, the analysis started as soon as possible. Additionally, 10% of all completed interviews were randomly selected for audio review by an independent quality auditor.

### Ethics approval and consent to participate

This study was conducted in accordance with the Declaration of Helsinki and approved by the Ethics Committee of Fujian Provincial Hospital (Approval No.: K2021-12-092; Date of Approval: December 30, 2021). Informed consent was obtained from all individual participants included in the study.

Written informed consent was obtained from all participants at the time of admission. Prior to the discharge interview, the investigators introduced themselves and re-obtained verbal consent from each participant.

### Study design

The following integrated services were provided during the hospitalization of patients.

**Project 1**. “Medical-nursing integration, whole process” responsibility-based nursing care.

Specific measures

(1) Each department improved the nursing process and nursing routines for all inpatients, including admission, examination, diagnosis and treatment, consultation, discharge, and follow-up visits. The service process was simplified, and the responsible nurse provided the entire physical and mental holistic nursing service for patients.
(2) The nurse's station was moved forwards to reduce the nursing care radius to within 3 meters of the patient. The responsible nurse accompanied the patient in his or her daily activities and communicated frequently with the patient.
(3) The responsible nurse followed the medical team in making ward rounds and participated in consultation for patients and discussion of difficult cases.

**Project 2**. Further improving the environmental management of homogeneous wards

Specific measures

(1) Morning and evening care were strictly implemented to ensure that the ward was clean and orderly.
(2) Noise in the departments was controlled, the wards were kept quiet and orderly, and noise was one of the special quality control inspection items.
(3) Attention was given to the organization of departmental housekeeping and keeping non-ward areas, such as treatment rooms, passages in wards, nurses' stations, doctor's offices, and medical duty rooms, clean and orderly.
(4) The materials on the walls of the wards were reviewed, and random posting was prohibited.
(5) The contents of the convenience service box were equipped according to the needs of specialists and inpatients. Stationery, needles and thread, straws, snacks, etc. were provided to meet the emergency needs of inpatients and their families. The departmental convenience service boxes were regularly replenished and updated.

**Project 3**. A book corner was set up in the ward to meet the reading needs of inpatients and their families, and the hospital's “Xinglin Bookstore” was built.

Specific measures

(1) A book corner was set up in the ward to provide health education materials, medical books, novels, newspapers, and periodicals for the convenience of inpatients and their families.
(2) Newspapers and periodicals were updated daily, and reading materials such as health education materials and books were updated regularly.

**Project 4**. The medical nurse–patient digital communication platform was optimized.

Specific measures

(1) Each ward established an optimized medical nurse–patient digital communication platform, such as a WeChat consultation group for medical staff and patients for specific diseases, a QQ group for patient communication, and an online consultation platform for patients, to provide extended care services.
(2) A patient message board was set up in each ward to solicit patients' suggestions and opinions. A special area for patient thank you letters was set up on the nurse–patient communication platform, and letters of praise from patients were pasted on the wall to pass on love and warmth.
(3) Nursing health education and publicity were digitized, health education is promoted, and nursing staff were organized to produce various forms of electronic education materials.

**Project 5**. Birthday and holiday blessings for inpatients

Specific measures

(1) The nursing department was responsible for making birthday cards according to the hospital's culture. Each department used the cards according to its actual demand.
(2) On patients' birthdays, the department filled in the patient's name on a greeting card and the head nurse, the medical team physician and the responsible nurse jointly sent a greeting card with wishes to the inpatients.
(3) Activities were conducted on major holidays, such as New Year's Day, Spring Festival, Dragon Boat Festival, Mid-Autumn Festival, National Day, and Double Ninth Festival, to send festive greetings to inpatients.
(4) Patients were organized to participate in festival activities held by the department, and blessings and warm wishes were sent to patients.

**Project** 6. Bedside settlement

Specific measures

(1) Admission and hospitalization procedures were completed quickly at the nurse's station or the one-stop patient service desk next to the ward, which saved patients and their families from traveling and increased convenience.

**Project** 7. “Didi Taxi” service in the hospital

Specific measures

(1) Internet-based hospitals provided wheelchair and flat transfer cart reservations. The “In-Hospital Didi Taxi” service reserved at the mobile terminal prepared more than 100 wheelchairs and flat transfer carts for free transfers to solve the issue of the “last 100 meters” after patients enter the hospital area.

Item 8. Peer education activities

Specific measures

(1) A peer education group was established, and patients with good self-management, good compliance, and strong communication skills were selected as core members.
(2) The nursing staff and the core team members jointly developed the peer education activity program and teaching content.
(3) A peer education activity was arranged once a month. The forms of activities included presentations (PPT), simulation teaching, and game-driven activities.

Project 9. Development of psychological nursing cases

Specific measures

(1) Psychological assessment and screening of admitted patients were conducted to screen high-risk patients. Nursing staff implemented personalized psychological nursing to patients under the guidance of nursing psychological theory and evidence.
(2) During hospitalization, the responsible nurse evaluated the mental status of patients with a focus on special groups of patients with previous psychological problems, preoperative and postoperative patients, patients with poor social support, older adult(s), and patients with cancer.

#### Themes and subthemes

The design of the interviews was based on the indicator database of patient experience from the National Medical Management Centre. Content validity was established through expert review, with item-level content validity indices ranging from 0.83 to 1.00 and a scale-level average content validity index of 0.94. Internal consistency was excellent, with a Cronbach's α of 0.94. The researchers identified three themes and 16 subthemes (Table 1). The three themes were (1) medical personnel, (2) logistic services, and (3) supporting facilities. These three themes were related to the service experience of patients receiving medical services during hospitalization, but the themes had different emphases (Table 1). At the end of the interviews, patients were invited to provide opinions and suggestions.

**Table 1:** Themes and subthemes.

Themes	Subthemes
Medical staff	1. Doctors respect
	2. The doctor listens
	3. Barrier-free communication between doctors
	4. Nurses
	5. Treat visitors
	6. Pain management
	7. First medication notification
	8. Health education
Logistics service	9. Medical technical examination
	10. Flat car wheelchair
	11. Care worker service
	12. Ward and toilet
	13. Road signs and instructions
	14. Meals
	15. Admission and discharge procedures
Overall rating	16. Overall experience score

The 5-point Likert responses (very satisfied, somewhat satisfied, generally satisfied, dissatisfied, very dissatisfied) were converted to numerical scores of 100, 80, 60, 40, and 20, respectively. The satisfaction score for each subtheme was calculated as the mean of the converted scores across all patients who responded to that subtheme. The overall satisfaction score for each year was the mean of the 16 subtheme scores. This linear conversion method is consistent with standard practice in Chinese hospital satisfaction surveys as recommended by the National Medical Management Centre, and has been validated in previous studies of patient satisfaction in China (7, 18).

Not all participants responded to every subtheme, as the applicability of certain services varied according to individual patient needs. Specifically, services such as caregiver services and pain management were only relevant to subsets of patients who actually received or utilized these services during hospitalization. Additionally, the evaluation of meal services and flat car wheelchairs applied only to patients who ordered hospital meals or used wheelchair services during their stay. Consequently, the sample size for each subtheme differs. The specific number of respondents for each subtheme in 2022 and 2023 is presented in Table 2. Overall, the response rate for most subtheme items approached 100% of the total sample, as these questions (e.g., doctor's respect, nursing service, ward and toilet) were applicable to all inpatients. However, due to their conditional applicability, the response rates for caregiver services and pain management were relatively lower, at approximately 53%. The response rates for wheelchair and meal services exceeded 70%. The variation in response rates across subthemes is inherent to the study design, as patients could only evaluate services they actually received. To ensure comparability, all subtheme satisfaction scores were calculated based only on responses from patients who had experienced that specific service. No imputation was performed for missing responses, as the non-response was due to inapplicability rather than random omission.

**Table 2:** The score of each subthemes of satisfaction.

Themes	Subthemes	Year	*n*	Scores	Mean standard error	*P-value*
Medical staff	1. Doctors respect	2022	22,800	98.96 ± 0.40	0.018	< 0.001
		2023	20,893	99.20 ± 0.84	0.039	
	2. The doctor listens	2022	22,800	98.87 ± 0.47	0.021	< 0.001
		2023	20,893	99.30 ± 0.82	0.039	
	3. Barrier-free communication between doctors	2022	22,800	98.74 ± 0.33	0.015	< 0.001
		2023	20,893	99.28 ± 0.96	0.045	
	4. Nursing service	2022	22,800	98.14 ± 2.08	0.094	< 0.001
		2023	20,893	98.77 ± 2.44	0.115	
	5.Treat visitors	2022	22,800	98.40 ± 1.76	0.079	0.028
		2023	20,893	98.66 ± 1.94	0.091	
	6. Pain management	2022	12, 540	98.07 ± 2.94	0.133	0.001
		2023	10,865	98.65 ± 2.21	0.104	
	7. First medication notification	2022	22,800	98.19 ± 1.90	0.086	0.282
		2023	20,893	98.32 ± 1.92	0.09	
	8. Health education	2022	22,800	97.69 ± 2.04	0.092	0.194
		2023	20,893	97.88 ± 2.32	0.109	
Logistics service	9. Medical technical examination	2022	22,800	98.44 ± 0.47	0.021	< 0.001
		2023	20,893	98.94 ± 1.06	0.05	
	10. Care worker service	2022	11,628	87.16 ± 3.12	0.141	< 0.001
		2023	11,073	88.65 ± 4.82	0.227	
	11. Meals	2022	16,188	93.02 ± 4.50	0.203	< 0.001
		2023	15,043	95.59 ± 3.88	0.183	
Supporting facilities	12. Admission and discharge procedures	2022	22,800	97.08 ± 2.36	0.107	0.001
		2023	20,893	97.63 ± 2.62	0.123	
	13. Flat car wheelchair	2022	15,960	96.22 ± 0.67	0.031	< 0.001
		2023	14,835	97.33 ± 1.69	0.08	
	14. Ward and toilet	2022	22,800	94.97 ± 3.54	0.16	0.013
		2023	20,893	94.35 ± 4.05	0.191	
	15. Road signs and instructions	2022	22,800	97.90 ± 2.94	0.133	0.175
		2023	20,893	98.13 ± 2.18	0.102	
	Total	2022	22,800	97.32 ± 1.72	0.078	0.887
		2023	20,893	97.34 ± 3.49	0.165	

*P* ≤ 0.05 was considered to be statistically significant.

### Data collection procedure

The researchers conducted structured telephone interviews using a computer-assisted telephone interviewing system. Each researcher sat in front of a computer wearing a headset, and the interview questionnaire was displayed on the screen, ensuring that all interviewers asked the exact same questions in the same order and with the same wording. The answers were directly entered into the computer system to minimize data entry errors.

To ensure data quality, the following standardized procedures were strictly enforced: (1) All interviewers completed a 4-h training session covering interview protocols, handling of refusal or non-response, and ethical considerations, followed by a certification test requiring 90% accuracy. (2) During the first 10 interviews of each interviewer, a senior researcher listened in real-time via a split-line connection and provided immediate feedback on any deviation from the protocol. (3) After each interview, the system automatically flagged responses that were incomplete (missing >10% of items) or illogical (e.g., very satisfied on all items but very dissatisfied overall), triggering an immediate call-back for clarification. (4) Within 24 h of each interview, a third-party quality auditor independently reviewed 10% of randomly selected call recordings and compared them against the entered data; the inter-rater agreement rate for data entry was consistently above 98%. (5) On the 3rd of each month, the hospital service centre exported all survey results from the previous month, and two independent reviewers cross-checked for anomalies; any discrepancy led to retrieval and re-review of the original recording.

### Statistical analysis

Data analysis was performed using SPSS version 23.0. Categorical variables were expressed as number(%) (including the number of samples and composition ratio). Continuous variables are expressed as the mean ± standard deviation (including the average score and overall score of satisfaction for each subtopic). The study did not set out to test a priori hypotheses; rather, its objective was to describe observed satisfaction levels across domains. Given the primary aim of this work—namely, to characterize satisfaction profiles across service domains and flag underperforming areas for internal quality use—a descriptive analytical framework was deemed appropriate. Mean scores, standard deviations, and between-year comparisons via *t*-tests were therefore employed as the mainstay of our quantitative reporting. This approach is consistent with that of earlier cross-sectional descriptive studies of patient satisfaction, in which the primary analytic goal was to describe satisfaction levels and identify demographic or service-related differences rather than to establish predictive models (10, 21). Similar descriptive frameworks have also been used in large-scale Chinese hospital satisfaction surveys aimed at quality monitoring and priority identification (7, 18). We acknowledge that multivariable modeling would offer additional insight into predictors of satisfaction, yet such analysis falls outside the prespecified scope of this quality assurance exercise. Instead, the present results are offered as a descriptive empirical basis from which more definitive, hypothesis-testing investigations may subsequently be designed.

## Results

### Participant information

This study interviewed a total of 43,693 discharged patients between 2022 and 2023. Table 3 provides the basic attributes of the participants. More than half of the participants, 61.76% in 2022 and 54.46% in 2023, were surgical patients. In 2022, the maximum number of participants was 9.91% in November and the minimum number was 6.68% in December. In 2023, the maximum number of patients was 10.86% in August and the minimum number was 4.23% in February.

**Table 3:** Characteristics of population distribution.

Characteristics	2022	2023
	*n* = 22,800 (100%)	*n* = 20,893 (100%)
Internal medicine	6,096 (26.74)	7,092 (33.94)
Department of surgery	14,081 (61.76)	11,378 (54.46)
Gynecology	1,425 (6.25)	1,386 (6.63)
Department of pediatrics	1,198 (5.25)	1,037 (4.96)
Month		
January	2,041 (8.95)	1,546 (7.40)
February	1,885 (8.27)	884 (4.23)
March	1,655 (7.26)	1,715 (8.21)
April	2,133 (9.36)	1,928 (9.23)
May	1,630 (7.15)	2,090 (10.00)
June	1,851 (8.12)	1,845 (8.83)
July	1,891 (8.29)	1,784 (8.54)
August	2,089 (9.16)	2,269 (10.86)
September	1,910 (8.38)	1,702 (8.15)
Oct.	1,934 (8.48)	2,043 (9.78)
November	2,259 (9.91)	1,838 (8.80)
December	1,522 (6.68)	1,249 (5.98)

The monthly data shown under “Month” are based on the total number of patients interviewed each month across all departments in the respective year.

### Themes

For nursing services to improve patients' experience in hospitals, the overall scores of the interview topics in 2022 and 2023 were 97.32 ± 1.72 and 97.34 ± 3.49 (*P* = *0.887*), respectively. In the interviews about the patient experience in 2022 and 2023, the three lowest-ranked satisfaction scores were (87.16 ± 3.12 vs. 88.65 ± 4.82; *P*<*0.001*) for caregiver services, (93.02 ± 4.50 vs. 95.59 ± 3.88; *P*<*0.001*) for meals, and (94.97 ± 3.54 vs. 94.35 ± 4.05; *P* = *0.013*) for wards and bathrooms. The satisfaction survey of doctor's respect, listening, nurse's service, pain management, examination, flat car wheelchair were all significantly improved (*P* < 0.001). There were no significant differences regarding satisfaction survey on medication notification, health education and road signs (*P* > 0.05; Table 2).

## Discussion and conclusion

### Discussion

The relationship between medical personnel and patients is fundamentally a healing relationship that involves mutual trust and mutual understanding as they work to jointly defeat the disease and alleviate pain. Poor nurse-patient relationship poses an obstacle to care delivery, jeopardizing patient experience and patient care outcomes (22). A patient-centred management philosophy should be formed to highlight the public welfare nature of public hospitals. The American College of Medicine defines “patient-centred” care as respecting and responding to the individual preferences, needs, and values of patients in clinical decision-making for medical services (23). The assessment of patients' medical experience and satisfaction can help medical institutions identify weak links in medical services, guide improvements in medical services, and promote patients' choices in medical treatment (24). The National Health Commission of China adheres to a people-centred approach. From 2015 to 2020, two consecutive rounds of action plans for further improvements in medical services were implemented to keep pace with the times and enhance the patient experience.

The average score for patient satisfaction in Fujian Province, China, in 2021 was 91.35 points. The overall self-examination scores in 2022 and 2023 were 97.32 ± 1.72 and 97.34 ± 3.49 (*P* = *0.887*), respectively, which were higher than the average levels. The total score of satisfaction in 2022 and 2023 was not statistically significant. This may be related to the fact that the hospital has been committed to the improvement of services and the overall patient experience of the hospital remains well. With the development of society, people's expectations of the medical industry have expanded beyond the treatment of diseases to include concern and care for the physical and mental health of patients. During hospitalization, patients pay attention to the quality of medical services and to their own experience and humanistic care. Studies have shown that hospital staff members' emotional support can affect patients' experience with the hospital (25). The service measure of sending good wishes on birthdays and holidays is a way to provide patients with real emotional support. Studies have shown patient experience with emotional support was the most significant dimension explaining patient satisfaction (18). Humanistic care responds to the psychological expectations of patients and aligns medical services with human nature. Studies have shown that patient satisfaction is closely related to high-quality treatment procedures (26). This finding is consistent with the results of our survey. Scores for doctor's respect, listening, nurse's service, pain management, examination, and flat car/wheelchair were significantly higher in 2023 compared with 2022 (*P* < 0.001). In the interviews, patients' general evaluation of the services of medical personnel was that they were able to answer questions in a timely manner and that the nurses smiled, were warm and friendly, and provided meticulous and rigorous nursing services. These findings indicate that patients' expectations of the hospital and medical personnel were met.

The persistently low satisfaction scores for caregiver services (approximately 53% response rate) and meals (response rate >70%). However, our satisfaction survey on logistic services showed that tertiary hospitals still have deficiencies in some areas. The satisfaction scores for caregiver services were (87.16 ± 3.12 vs. 88.65 ± 4.82; *P*<*0.001*), for meals (93.02 ± 4.50 vs. 95.59 ± 3.88; *P*<*0.001*).There was a statistically significant difference between 2022 and 2023. The observed score increases suggest a possible positive association with the implemented service measures, although causal attribution cannot be established. Regarding the subthemes, caregivers and the meal experience scored low. Some patients said that the meals in the canteen were too bland and had little variety. From the perspective of expectation-disconfirmation theory, these domains likely suffer from high patient expectations that are not met by current service provision. Unlike clinical services where patients may have limited prior knowledge, food and caregiving are everyday life experiences with which all patients have well-established expectations. The gap between home-cooked meals or family-provided care and institutional food or third-party caregiver services is inherently larger. Sometimes the food in the canteen varied in saltiness. Ancillary departments such as testing, and examinations were slow to perform checks and had a long waiting time. In terms of supporting facilities, patients said that the hospital had advanced and complete diagnostic and treatment equipment, but some supporting facilities needed to be improved, such as long waiting times for the elevator, outdated ward facilities, and an imperfect electronic reminder system. Patients' experience with the hospital environment was mostly negative. Items that patients mentioned the most included old wards, poor hygienic conditions, and loud noise. A great opportunity is there to improve patient's satisfaction level if the service quality is improved around the time of patient and health care provider interaction and facility amenity services (21). Improving the hospital environment is an important measure to improve hospital satisfaction. This is consistent with our previous findings (7). It should be emphasized that public tertiary hospitals are already the highest-level hospitals in China. As a Class A tertiary medical institution in China, the researcher's hospital owns and uses a large amount of advanced equipment, in line with the high expectations of patients. Due to the large number of patients and their needs, the sanitation facilities and comfort of the hospital cannot be maintained, resulting in high levels of environmental noise and unsatisfactory hygienic status.

The feedback from the patient experience survey allowed the researchers to understand the needs and expectations of patients, including their physiological, psychological, and social needs. Medical institutions continue to improve comprehensive service measures to enhance the patient experience, and the hospital environment affects this experience. We aim to create a comfortable and humanized medical environment, including the design of the ward and the layout of medical equipment. Medical processes are optimized, patients are prioritized, waiting time is reduced, and convenient online appointment and registration services are provided to improve efficiency. Patients' autonomy is respected, patient education programmes are conducted, relevant information and support are provided, patients are involved in treatment decisions and treatment plans, and patients' wishes and needs are fully considered. Studies have shown that empowering patients can improve their outcomes, satisfaction, and treatment adherence (27). Furthermore, an app is used as a food ordering service. If the treatment diet is met, patients can choose dishes according to their personal preferences. Studies have shown that hospital food service is a key patient experience domain in an inpatient setting, which also plays an important role in responding to clinical and nutritional needs by providing food that is acceptable to patients (28). The hospital has established a patient feedback mechanism to understand patients' opinions and suggestions in a timely manner. If a complaint is received from a patient, the following steps are taken: (1) listen carefully and confirm the complaint; (2) express sympathy and a sincere apology; (3) take action to compensate for the error; (4) provide an explanation to clear up the misunderstanding and confirm that the other party is satisfied; and (5) summarize for improvement.

The interview results showed that patients gave the lowest score to their experience with the caregiver service. This may be related to the fact that the caregivers are personnel stationed in the hospital by a third-party organization, and the quality of the personnel varies. Therefore, medical institutions should strengthen caregivers' training on the concept of service and provide jobs to individuals who pass an assessment. The training content includes the following aspects. First, we must establish a correct concept of service by providing attentive service to patients from the moment they walk in. Warmth and friendliness can significantly improve the patient experience. Second, the service behaviors of dressing standards for caregivers and service standardization are regulated. Treating others with sincerity and empathy can create a sense of trust in patients. Third, communication skills should be mastered. Patients need to adapt to change from a social role to a patient role. They may feel strange, uncomfortable and anxious about the hospital. Some interviewees said that they lacked effective communication with the hospital staff, and they sometimes could not communicate well due to misunderstandings of tone, insufficient communication time, and a lack of professional language. To address these problems, it is necessary to pay attention to the rational use of language, voice intonation, and appropriate body language. How someone says something is often more important than what is said. It is necessary to adopt two avoidances and three attentions. The two forms of avoidance include, first, avoiding the use of language and tone that can irritate the patient, preventing disrespect by using imperative language, and forbidding personal insults, and second, avoiding the excessive use of professional vocabulary, which can increase patients' anxiety and cause negative associations. The three kinds of attention are attention to the patient's education level and feelings about communication; the patient's disease cognition and knowledge; and the emotional responses of the patient and the medical staff. Self-control should be used when necessary, and attempts should be made to comfort and guide patients. Fourth, when complaints are received, measures should be taken to carry out service recovery. Studies have shown that positive communication behavior and trust-building behavior are significantly positively correlated with patient satisfaction (29).

Informatization is a way for hospitals to improve medical services. Informatization involves the use of the internet and technology for whole-process business process integration. “Seeing a doctor with a smartphone in hand” is a type of informatization. The starting point is to take every patient seriously and provide patients with the best treatment and with comfortable and convenient service. Supporting facilities include the following. (1) The hospital has established a one-stop service centre to accept patient complaints. With the help of digitized means, the hospital has also taken the lead in implementing the “Didi Taxi” service, which can be booked on a mobile terminal. Reservations for wheelchairs and flat transfer carts are provided, and the hospital provides services as soon as possible after patients arrive at the hospital according to the time, period, and location of appointments to achieve seamless connections. (2) The bedside settlement has been introduced throughout the hospital. Services such as admissions and discharge were moved to the bedside in the ward so that these procedures can be completed at the nurse's station in the ward, which significantly reduces processing time. Patients can go use one-stop services, such as admission registration, payment and settlement, and list printing, in the ward. The system automatically sends a text message to remind the patient to go to the nursing station for the discharge process, which takes 5 min to complete. The nurses hand over medications and receipts to the patient during discharge. The participants stated that this approach could reduce the time for patients to travel back and forth and could turn a “waiting service” into an “active service.” Patients can follow the hospital's official account on their mobile phones to access nearly 50 functions, such as appointment registration, top-up and payment, report query, satisfaction surveys, and information through the “Handheld Hospital” platform to provide patients with fast and convenient medical services. Solving practical problems in this process can improve patients' experience and satisfaction. (3) In-hospital intelligent navigation and guidance services have been realized with the help of Internet of Things technology and intelligent robots. Based on Internet of Things technology, an in-hospital navigation service combined with the medical treatment process has been launched that provides functions such as the sharing of real-time locations and parking and clock-in. This service is combined with intelligent robots to build an indoor intelligent navigation and diagnosis service system in hospitals. From the moment a patient enters the hospital area, the parking location and the visiting department are all clear. The navigation path can be accurate within three steps to effectively meet patients' urgent needs to “find a place, find a person, and find a car.” (4) An intelligent hospital-wide examination appointment platform has been constructed. This system automatically uses an intelligent rules engine to complete payment and make appointments. It takes only 1 min to push all the information, including the examination appointment time, examination location and precautions, to the patient, solving the problem of patient queuing and errands and shortening the waiting time. (5) Health education is provided. Departments are equipped with iPads that automatically import basic patient information, such as telephone numbers and admission and exit information, through the hospital's HIS system. Nurses maintain health education content, which includes the ward environment, health education on admission, and disease education. This content can be in the form of texts, pictures, and video links. Automatic receiving of objects and content as well as the receiving time and frequency can be set. Studies have shown that nursing managers develop nursing practice standards and educational programs would improve patient experiences (30). When a patient is admitted to or discharged from the hospital, the responsible nurse clicks the iPad and automatically sends a text message through the hospital's text message platform to remind the patient to read the relevant information. An official WeChat account for the hospital or department has been established. Patients can follow this account during admission to the hospital, and health education content can be regularly released so that patients can obtain relevant information. Paper health education leaflets are placed in the waiting area and wards of the hospital so that patients can access them at any time.

#### Policy implications

Based on our findings, we propose the following actionable recommendations for hospital administrators and policymakers: (1) Caregiver service reform: establish hospital-supervised training and certification programs for third-party caregivers, including mandatory communication skills training and patient feedback mechanisms linked to agency contracts. (2) Meal service improvement: implement digital ordering systems allowing patients to select meals from approved menus while meeting nutritional requirements; consider decentralized kitchen models to reduce food transport time. (3) Environmental upgrades: prioritize renovation of ward bathrooms and noise reduction measures (e.g., replacing rolling carts with silent wheels, installing soft-closing doors). (4) Scalability of digital innovations: The “Didi Taxi” wheelchair reservation and bedside settlement systems demonstrated strong patient acceptance and should be considered for nationwide scaling in tertiary hospitals. (5) Monitoring framework: Establish a real-time dashboard tracking subtheme-level satisfaction scores by department, enabling early identification of deteriorating service domains before they impact overall satisfaction.

Before closing, a caveat warrants restatement: our analytical strategy was deliberately kept descriptive, reflecting the study's quality-surveillance orientation. This obviously constrains causal interpretation. Even so, the consistent identification of caregiving and catering as persistently low-scoring areas—coupled with observable gains in doctor-patient communication and nursing domains—does furnish actionable intelligence for managerial decision-making. Whether these improvements are directly attributable to the integrated service package, however, awaits confirmation in controlled, multi-centre work. Thus, the year-to-year differences reported here should be interpreted as descriptive trends that may inform hypothesis generation, rather than as evidence of intervention effectiveness.

## Conclusion

Patients can be provided with a good medical experience through service measures. The quality of medical service is the key to improving the patient experience. By focusing on patients' needs, emotions and experiences, we may provide comprehensive care and support and truly respond to the “patient-centred” concept of medical service, which may improve not only medical quality but also the doctor–patient relationship and promotes the development of the medical industry.

### Limitations of the study

This study has several limitations. First, this survey used convenience sampling. The interviewees were divided according to the department in which they were hospitalized but not by the type or severity of disease. The departments excluded from the survey included the cadre ward, the delivery ward, the ICU, and the day ward, which may limit the generalizability of our findings to these patient populations. To mitigate sampling bias, we used almost the same proportion of the total number of patients discharged from each department each month to conduct interviews, and patients were shuffled and sorted according to discharge time to reduce data bias.

Second, we were unable to capture or adjust for individual-level sociodemographic characteristics of respondents—such as gender, age, education level, occupation, or disease severity—all of which are known to influence patients' perceptions of care and satisfaction. The absence of these variables precluded stratification or covariate adjustment, and may therefore affect the interpretability of the observed between-group or between-year comparisons.

Third, because hospitalization stays were relatively short, not every inpatient could experience all the services covered in our survey. This resulted in varying denominators across subtheme items (see Table 2), with conditional services such as caregiver support and pain management having response rates of approximately 53%. While this variation is inherent to service-specific evaluation and does not compromise the internal validity of each subtheme estimate, it limits direct comparability of satisfaction scores across domains with markedly different respondent compositions.

Fourth, our analysis was deliberately kept descriptive—employing means, standard deviations, and *t*-tests—in keeping with the study's primary quality-surveillance objective of characterizing satisfaction patterns and flagging low-scoring domains for internal action. We acknowledge, however, that the absence of multivariable regression modeling precludes adjustment for potential confounders (including the unmeasured sociodemographic factors noted above) and does not allow us to isolate the independent contribution of any single service component to overall satisfaction. Consequently, the observed satisfaction scores and year-to-year differences should be read as descriptive trends rather than causal or adjusted effect estimates. Similar analytical choices and their attendant limitations have been noted in other large-scale hospital satisfaction surveys (7, 10, 21), where descriptive and bivariate analyses were used to map satisfaction patterns and inform quality improvement priorities. Future investigations with larger, multi-centre samples should employ multivariable regression models to adjust for patient-level confounders and to isolate the independent effects of specific service domains on overall satisfaction.

Fifth, this was a single-centre investigation, which may constrain the generalizability of our findings to other hospital settings or geographic regions. Future work should consider multi-centre designs, incorporate random or stratified sampling, and apply regression-based approaches with fuller case-mix adjustment to validate and extend the descriptive observations reported here.

### Relevance to clinical practice

Due to human biological attributes, we will all face health problems in our life and play the role of “patients.” With the development of modern medicine, medical personnel have paid increasing attention to humanistic care. However, satisfaction is just the starting point, and service never ends. We are committed to providing patients with high-level medical services with convenient processes, efficient operation, and symmetric information. Improving the medical experience is not only about personal feelings but is also a reflection of civilization and the progress of society.

Parts of this work were presented as a poster at the International Council of Nurses Congress 2025, held in Helsinki, Finland, in June 2025.

## Data Availability

The raw data supporting the conclusions of this article will be made available by the authors, without undue reservation.

## References

[1] Karaca A Durna Z. Patient satisfaction with the quality of nursing care. Nurs Open. (2019) 6:535–45. doi: 10.1002/nop2.237 30918704 PMC6419107

[2] Zhang Y Li Q Liu H. From patient satisfaction to patient experience: a call to action for nursing in China. J Nurs Manag. (2020) 28:450–6. doi: 10.1111/jonm.12922 31793108

[3] Chung C McKenna L Cooper SJ. Patients' experiences of acute deterioration: a scoping review. Int J Nurs Stud. (2020) 101:103404. doi: 10.1016/j.ijnurstu.2019.103404 31670222

[4] Beckett MK Quigley DD Lehrman WG Giordano LA Cohea CW Goldstein EH . Interventions and hospital characteristics associated with patient experience: an update of the evidence. Med Care Res Rev. (2024) 81:195–208. doi: 10.1177/10775587231223292 38238918

[5] Wang J Kidd VD Giafaglione B Strong B Ohri A White J . Improving nurse-physician bedside communication using a patient experience quality improvement pilot project at an academic medical center. Cureus. (2024) 16:e55976. doi: 10.7759/cureus.55976 38469366 PMC10927320

[6] Fang J Liu L Fang P. What is the most important factor affecting patient satisfaction - a study based on gamma coefficient. Patient Prefer Adher. (2019) 13:515–25. doi: 10.2147/PPA.S197015 31114168 PMC6489650

[7] Zhou F Xu C Sun Y Meng X. Influencing factors of outpatients' satisfaction in China a cross-sectional study of 16 public tertiary hospitals. Patient Prefer Adher. (2021) 15:1243–58. doi: 10.2147/PPA.S311786 34135576 PMC8200138

[8] Joshi A Shrestha D. Surgical service utilization and in-patient satisfaction in surgical service. J Nepal Health Res Counc. (2024) 21:667–71. doi: 10.33314/jnhrc.v21i4.5011 38616600

[9] Adhikari M Paudel NR Mishra SR Shrestha A Upadhyaya DP. Patient satisfaction and its socio-demographic correlates in a tertiary public hospital in Nepal: a cross-sectional study. BMC Health Serv Res. (2021) 21:135. doi: 10.1186/s12913-021-06155-3 33579283 PMC7881603

[10] Alharbi HF Alzahrani NS Almarwani AM Asiri SA Alhowaymel FM. Patients' satisfaction with nursing care quality and associated factors: a cross-section study. Nurs Open. (2023) 10:3253–62. doi: 10.1002/nop2.1577 36585398 PMC10077356

[11] He X Li L Bian Y. Satisfaction survey among primary health care outpatients in the backward region: an empirical study from rural Western China. Patient Prefer Adher. (2018) 12:1989–96. doi: 10.2147/PPA.S172021 30323568 PMC6173270

[12] Desta H Berhe T Hintsa S. Assessment of patients' satisfaction and associated factors among outpatients received mental health services at public hospitals of Mekelle Town, northern Ethiopia. Int J Ment Health Syst. (2018) 12:38. doi: 10.1186/s13033-018-0217-z 30008801 PMC6042256

[13] Yu W Li M Xue C Wang J Liu J Chen H . Determinants and influencing mechanism of outpatient satisfaction: a survey on tertiary hospitals in the People's Republic of China. Patient Prefer Adher. (2016) 10:601–12. doi: 10.2147/PPA.S104453 27143865 PMC4844439

[14] Tevis SE Kennedy GD Kent KC. Is there a relationship between patient satisfaction and favorable surgical outcomes? Adv Surg. (2015) 49:221–33. doi: 10.1016/j.yasu.2015.03.006 26299501 PMC4548286

[15] Grone O Garcia-Barbero M. Services WHOEOfIHC. Integrated care: a position paper of the WHO European Office for Integrated Health Care Services. Int J Integr Care. (2001) 1:e21. doi: 10.5334/ijic.28 16896400 PMC1525335

[16] Zhang J Yang L Wang X Dai J Shan W Wang J. Inpatient satisfaction with nursing care in a backward region: a cross-sectional study from northwestern China. BMJ Open. (2020) 10:e034196. doi: 10.1136/bmjopen-2019-034196 32912940 PMC7482479

[17] Davins J Gens M Pareja C Guzman R Marquet R Valles R. [Catalonia's primary healthcare accreditation model: a valid model]. Med Clin. (2014) 1:74–80. doi: 10.1016/j.medcli.2014.07.015 25128364

[18] Chen X Yuan J Zhao W Qin W Gao J Zhang Y. Which aspects of patient experience are the 'moment of truth' in the healthcare context: a multicentre cross-sectional study in China. BMJ Open. (2024) 14:e077363. doi: 10.1136/bmjopen-2023-077363 38326249 PMC10860089

[19] Rahman MK Bhuiyan MA Zailani S. Healthcare services: patient satisfaction and loyalty lessons from Islamic friendly hospitals. Patient Prefer Adher. (2021) 15:2633–46. doi: 10.2147/PPA.S333595 34866903 PMC8633710

[20] McGrath C Palmgren PJ Liljedahl M. Twelve tips for conducting qualitative research interviews. Med Teach. (2019) 41:1002–6. doi: 10.1080/0142159X.2018.1497149 30261797

[21] Asamrew N Endris AA Tadesse M. Level of patient satisfaction with inpatient services and its determinants: a study of a specialized hospital in Ethiopia. J Environ Public Health. (2020) 2020:2473469. doi: 10.1155/2020/2473469 32855641 PMC7443030

[22] Feng Y Liu C Tao S Wang C Zhang H Liu X . Developing and validating the nurse-patient relationship scale (NPRS) in China. BMC Nurs. (2024) 23:255. doi: 10.1186/s12912-024-01941-w 38649929 PMC11034141

[23] Handley SC Bell S Nembhard IM. A systematic review of surveys for measuring patient-centered care in the hospital setting. Med Care. (2021) 59:228–37. doi: 10.1097/MLR.0000000000001474 33229897 PMC7878319

[24] Algudairi G Al-Eisa ES Alghadir AH Iqbal ZA. Patient satisfaction with outpatient physical therapy in Saudi Arabia. BMC Health Serv Res. (2018) 18:888. doi: 10.1186/s12913-018-3646-0 30477495 PMC6258285

[25] Wang X Chen J Burstrom B Burstrom K. Exploring pathways to outpatients' satisfaction with health care in Chinese public hospitals in urban and rural areas using patient-reported experiences. Int J Equity Health. (2019) 18:29. doi: 10.1186/s12939-019-0932-3 30728005 PMC6366112

[26] Tsai TC Orav EJ Jha AK. Patient satisfaction and quality of surgical care in US hospitals. Ann Surg. (2015) 261:2–8. doi: 10.1097/SLA.0000000000000765 24887985 PMC4248016

[27] Cao F Hong F Ruan Y Lin M. Effect of patient-empowerment interaction model on self-management ability of peritoneal dialysis patients: a randomized controlled trial. Patient Prefer Adher. (2023) 17:873–81. doi: 10.2147/PPA.S402698 37009428 PMC10065006

[28] Abid MH Shehri NA Din S Nofeye JA. Leveraging an experience-based codesign approach to improve the inpatient food service experience. Glob J Qual Saf Healthc. (2023) 6:89–95. doi: 10.36401/JQSH-23-2 38405328 PMC10887477

[29] Tang C Tian B Zhang X Zhang K Xiao X Simoni JM . The influence of cultural competence of nurses on patient satisfaction and the mediating effect of patient trust. J Adv Nurs. (2019) 75:749–59. doi: 10.1111/jan.13854 30209816

[30] Kang HJ Yu S. Nurses' perspectives of the patient care experience assessment items using importance-performance analysis. J Nurs Manag. (2022) 30:3247–55. doi: 10.1111/jonm.13755 35939348

